# scLTNN: an innovative tool for automatically visualizing single-cell trajectories

**DOI:** 10.1093/bioadv/vbaf033

**Published:** 2025-02-26

**Authors:** Cencan Xing, Zehua Zeng, Lei Hu, Jianing Kang, Shah Roshan, Yuanyan Xiong, Hongwu Du, Tongbiao Zhao

**Affiliations:** Daxing Research Institute, School of Chemistry and Biological Engineering, University of Science and Technology, Beijing, Beijing 100083, China; Daxing Research Institute, School of Chemistry and Biological Engineering, University of Science and Technology, Beijing, Beijing 100083, China; Daxing Research Institute, School of Chemistry and Biological Engineering, University of Science and Technology, Beijing, Beijing 100083, China; School of Life Sciences, Westlake University, Hangzhou 310030, China; Daxing Research Institute, School of Chemistry and Biological Engineering, University of Science and Technology, Beijing, Beijing 100083, China; Daxing Research Institute, School of Chemistry and Biological Engineering, University of Science and Technology, Beijing, Beijing 100083, China; Key Laboratory of Gene Engineering of the Ministry of Education, Institute of Healthy Aging Research, School of Life Sciences, Sun-Yat-sen University, Guangzhou 510006, China; Daxing Research Institute, School of Chemistry and Biological Engineering, University of Science and Technology, Beijing, Beijing 100083, China; State Key Laboratory of Stem Cell and Reproductive Biology, Key Laboratory of Organ Regeneration and Reconstruction, Institute of Zoology, Chinese Academy of Sciences, Beijing 100101, China; Beijing Institute for Stem Cell and Regenerative Medicine, Beijing 100101, China

## Abstract

**Motivation:**

Cellular state identification and trajectory inference enable the computational simulation of cell fate dynamics using single-cell RNA sequencing data. However, existing methods for constructing cell fate trajectories demand substantial computational resources or prior knowledge of the developmental process.

**Results:**

Here, based on the discovery of the consistent expression distribution of highly variable genes, we create a new tool named scRNA-seq latent time neural network (scLTNN) by combining an artificial neural network with a distribution model. This innovative tool is pre-trained and capable of automatically inferring the origin and terminal state of cells, and accurately illustrating the developmental trajectory of cells with minimal use of computational resources and time. We implement scLTNN on human bone marrow cells, mouse pancreatic endocrine lineage, and axial mesoderm lineage of zebrafish embryo, accurately reconstructing their cell fate trajectories, respectively. Our scLTNN tool provides a straightforward and efficient method for illustrating cell fate trajectories, applicable across various species without the need for prior knowledge of the biological process.

**Availability and implementation:**

https://github.com/Starlitnightly/scLTNN.

## 1 Introduction

Computational simulation of cell fate dynamics offers unique opportunities to gain insights into various biological processes, including disease evolution, life development, cellular regulation, and more (Bendall *et al.* 2014, Griffiths *et al.* 2018, Kulkarni *et al.* 2019). Single-cell transcriptomics allows for profiling cellular state on transient slices along cell fate trajectories, such as the origin and end fate of any cell at a single-cell resolution (Bergen *et al.* 2020). Computational analysis of single-cell transcriptome data enables the prediction of cell states and their sequence along developmental trajectories, facilitating cell state identification and trajectory inference (Qiu *et al.* 2017, Saelens *et al.* 2019). Algorithms for trajectory inference, such as the transcriptional dynamics-based RNA velocity algorithms that relies on the ratio of spliced-to-unspliced mRNA (Bergen *et al.* 2020, Lange *et al.* 2022), and the graph-based evolutionary algorithms that depends on the expression of key genes (Cao *et al.* 2019, Wolf *et al.* 2019), have the ability to reorder cells in heterogeneous states and allocate them to ordered trajectories (Haghverdi *et al.* 2016, Setty *et al.* 2016). The RNA velocity-based algorithm requires a large number of computational resources and time; whereas the key gene-based graph algorithm relies on artificial selection of developmental root and direction that depends on *a priori* knowledge (Wolf *et al.* 2019). These limiting factors restrict their use in certain settings, particularly those with highly heterogeneous cellular populations like cancer cells.

**Figure 1.**
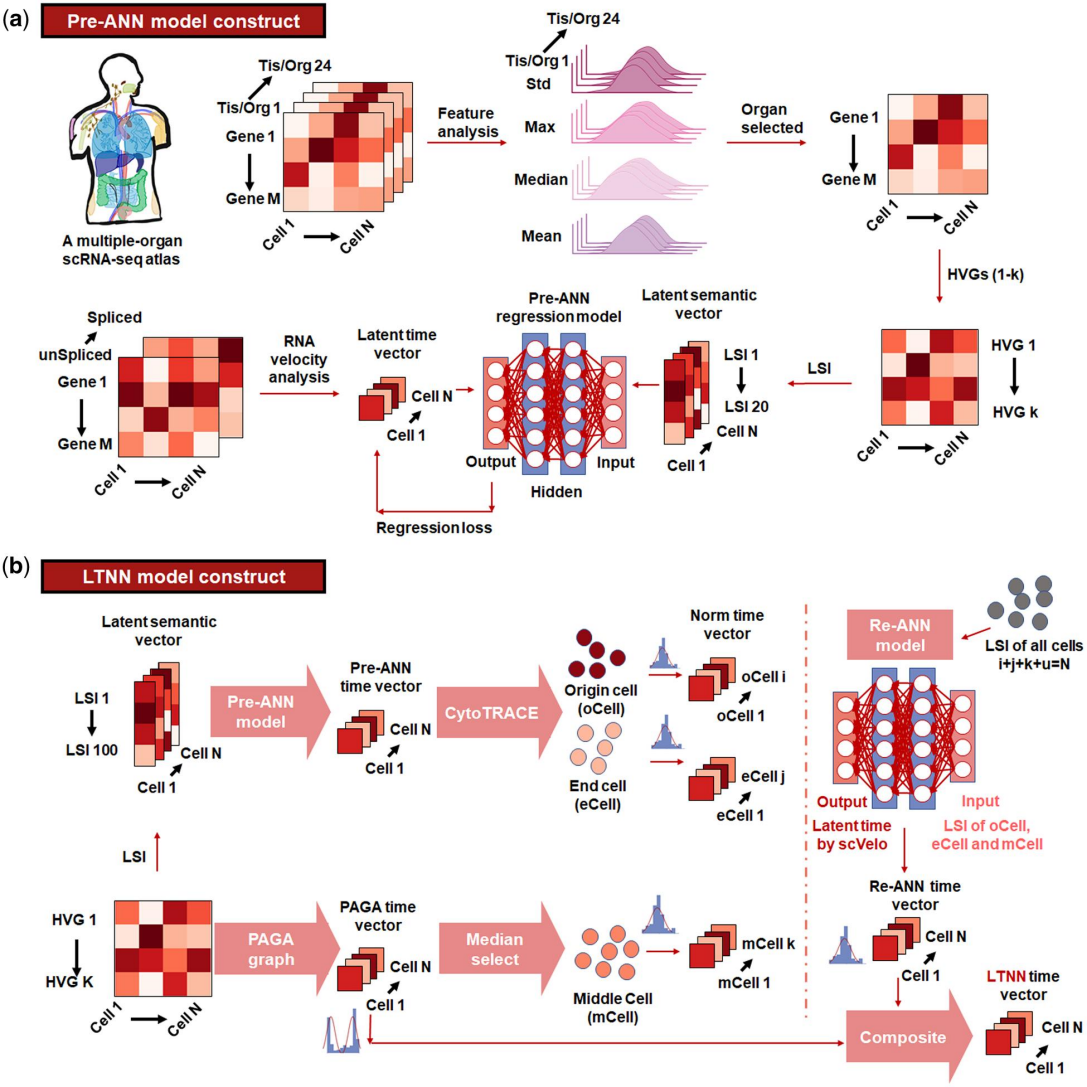
Overview of the scLTNN model. (a) Pre-ANN model construct. The construction of the Pre-ANN model begins with the original scRNA-seq data of 24 human tissues and organs, obtained from a recent publication (Tabula Sapiens Consortium *et al.* 2022). Each tissue/organ contains *N* cells and *M* genes. Initially, the distribution features of gene expression in each organ are analyzed, and genes with similar expression features are filtered out. The remaining HVGs (1−*k*) of cells (1−*N*) in each tissue/organ are then assigned values of the LSI, resulting in latent semantic vectors. These latent semantic vectors with LSI values are set as the input for Pre-ANN regression. Concurrently, the latent time vectors, generated by VeloVI based on the unspliced and spliced mRNA matrix of the same organ, are set as the output for regression. Ultimately, the Pre-ANN model is trained through loss evaluation. (b) LTNN model construct. Initially, the origin and end cell clusters are determined using the pre-ANN time vector, which is regressed by the pre-ANN model and further refined by CytoTRACE according to gene expression. Subsequently, the middle cell cluster is determined by the PAGA time vector, constructed using the HVG matrix and origin cells. The Re-ANN model is then constructed, using the LSI of origin, middle, and end cells as input, and the latent time of these cells, calculated by VeloVI, as output. Finally, the LTNN model is obtained by integrating the statistical distributions of Re-ANN time and PAGA time. Tis/Org, tissues and organs; Std, standard deviation; Max, maximum value; Median, median value; Mean, mean value; HVGs, high-variable genes; LSI, Latent Semantic Index; Pre-ANN, the pre-trained artificial neural network model; Re-ANN, the repeated-ANN model.

To improve the efficiency and accessibility of trajectory inference, we create a modular framework known as scRNA-seq latent time neural network (scLTNN). This approach builds on a pseudotime algorithm, LTNN, which utilizes a multi-organ pre-trained model to predict cell development trajectories without relying on prior knowledge or consuming significant computational resources and time. Our work demonstrated the similarity of developmental processes across multiple organs within a single species using artificial neural networks (ANN), marking a pioneering step in characterizing multi-organ development. The scLTNN generally involves the following steps: First, a pre-trained artificial neural network model (Pre-ANN) is built based on the latent time calculated by RNA velocity (Fig. 1a). For the same species and the same sequencing platform, the model only needs to be trained once on a tissue or organ. We have provided Pre-ANN models for humans, zebrafish, and mice. Second, the origin and end cell states are automatically identified from Pre-ANN model and corrected by CytoTrace value (Gulati *et al.* 2020), then middle cells are identified using diffusion graph (Fig. 1b). scLTNN models the normal distribution of cells in origin, middle and terminal, then could train the repeated-ANN (Re-ANN) model and use the trained Re-ANN model to predict the pseudotime of the other cells. Finally, we sample the predicted Re-ANN time using a double Weber distribution, sample the diffusion pseudotime (DPT) using a normal distribution, and merge the two values to finally obtain the LTNN time (Fig. 1b).

## 2 Methods

### 2.1 Determining the distribution features of HVGs across various human/mouse tissues and organs

The scRNA-seq data from 24 human tissues and organs were downloaded from a recent publication (Tabula Sapiens Consortium *et al.* 2022). The scRNA-seq data from 17 mouse tissues and organs were downloaded from a recent publication (Schaum *et al.* 2018). The data was quantified and normalized by Omicverse, and the top 10 000 highly variable genes (HVGs) were analyzed using Pearson (Wolf *et al.* 2018). Following the determination of the maximum, mean, and median expression of genes in each cell, the distribution of maximum, mean, and median expression in each tissue and organ was visualized using histogram plots. Standard deviation was employed to assess the variability in the distribution of maximum, mean, and median expression across cells. The resulting data were deposited at Figshare (Zeng 2023).

### 2.2 Preparing the LSI vector

The raw data from 24 human tissue and organs (GSE201333)(Tabula Sapiens Consortium *et al.* 2022) and 17 mouse tissue and organs (GSE109774) (Schaum *et al.* 2018), the human bone marrow (https://data.humancellatlas.org/explore/projects/091cf39b-01bc-42e5-9437-f419a66c8a45) (Saelens *et al.* 2019), mouse pancreatic endocrine lineage (GSE132188)(Bastidas-Ponce *et al.* 2019), and the axial mesoderm lineage of zebrafish embryo (GSE106587) (Farrell *et al.* 2018) were retrieved from the Gene Expression Omnibus repository in the National Center for Biotechnology Information. Cells with low sequencing counts (<1000) and a high mitochondrial fraction (>0.2) were excluded in further analysis. The filtered count matrix was normalized by dividing the counts of each cell by total molecules counts detected in that particular cell. The top 10 000 HVGs were identified using the Seurat_v3 function in the Python library scanpy (Wolf *et al.* 2018) and omicverse (Zeng *et al.* 2024). The non-high-variable genes (nHVGs) were determined by intersecting genes that recur in 10% of the cells in 24 human tissues and organs and nHVGs in scRNA-seq.

### 2.3 Implementing LSI within the Pre-ANN and Re-ANN

The scRNA-seq data from different tissues and organs were processed using latent semantic indexing (LSI), with each scRNA-seq dataset comprising m cells and each cell containing n genes. The matrix prepared for LSI analysis is denoted $A_{m\times n}$ (Hofmann 1999). The Singular Value Decomposition (SVD) was employed to break down the matrix $A_{m\times n}$ into three distinct matrices:

$$A_{m\times n}=A_{m\times m}\sum_{m\times n}V_{n\times n}^{T}$$

Assuming the presence of k topics, rank reduction was performed on the SVD decomposition:

$$A_{m\times n}\approx A_{m\times k}\sum_{k\times k}V_{k\times n}^{T}$$

For the scRNA-seq data, $t_{m}$ represented the timing sequence (pseudotime) of the cells, which was also considered as a latent semantic. Using the matrix $A_{m\times k}\;$and $t_{m}$, a regression model was constructed. The LSI was calculated using the omicverse library in Python.

We assumed *K* (*K* = 5) layers in the ANN (Wang 2003). ${\mathrm{net}}^{\left(K\right)}$ represents all nodes in the *K* layer. $Y^{\left(K\right)}$ denotes each layer’s output. To train the regression model, we minimize the following objective function:

$$\begin{array}{c} L=\frac{1}{2}\int_{i=1}^{d_{K}}{\left(Y_{i}^{\left(K\right)}-T_{i}\right)}^{2}\\ Y_{i}^{\left(K\right)}=f_{i}^{\left(K\right)}\left({\mathrm{net}}_{i}^{\left(K\right)}\right)=W_{i}^{\left(K\right)}Y_{i}^{\left(K-1\right)}+b_{i}^{\left(K\right)} \end{array}$$

 $W_{i}^{\left(K\right)}$ represents the weight of node i in *K*th layer, while $b_{i}^{\left(K\right)}$ is the bias of node i in the same layer. The active function in neuron, denoted by $f_{i}^{\left(K\right)}$, uses the Rectified Linear Unit (ReLu) in the hidden layer and Linear in the output layer. The minimization of the objective function is achieved through gradient-based optimization using the PyTorch library in Python. To assess the consistency between the regression result ${\hat{y}}_{i}$ and the true result $y_{i}$, the mean absolute error (MAE) is used.

$$\mathrm{MAE}=\frac{1}{n}\sum_{i=1}^{n}\left|\left({\hat{y}}_{i}-y_{i}\right)\right|$$

The ANN structure of Pre-ANN and Re-ANN are the same, except that Re-ANN model only uses the states of origin, middle and end cell to train the model.

### 2.4 Training datasets for Pre-ANN model

For the Pre-ANN model, the detailed computational steps have been fully described in the previous section, and here we present the sources of training data for three different species. Note that the Pre-ANN model needs to be re-trained for different means of library construction and for different species; see https://scltnn.readthedocs.io/en/latest/Tutorials/model_construst.html for a training tutorial.

Human bladder cells:

https://figshare.com/articles/dataset/Tabula_Sapiens_RNA_velocity/17065466

Mouse dentate Gyrus neurogenesis cells:

https://scvelo.readthedocs.io/en/stable/scvelo.datasets.dentategyrus.html

Zebrafish neural crest-derived cells from trunks:

https://dynamo-release.readthedocs.io/en/latest/notebooks/zebrafish.html

All cell dataset of these three species were from drop-seq based on 10× Genomics.

### 2.5 Determining of cell states

To identify the various cell states, we utilized a primary ANN model to predict the latent time for each cell. The process begins with the computation of ANN time $t_{\mathrm{ANN}}\;$for each cell i, represented as:

$$t_{\mathrm{ANN}}(i)=\mathrm{PreANN}(X_{\mathrm{lsi}}(i))$$

where $X_{\mathrm{lsi}}(i)$ represents the input data for cell i in the LSI space

To ensure the correct orientation of the ANN time, we utilized the CytoTRACE, which uses the number of genes expressed per cell as a signal of differentiation status. Specifically, we calculated the mean expression of genes for cells with low and high predicted times:

$$\begin{array}{c} \mathrm{Cytotrac}e_{\mathrm{low}}=\frac{1}{N_{\mathrm{low}}}\sum_{i\in I_{\mathrm{low}}}(E(i))\\ \mathrm{Cytotrac}e_{\mathrm{high}}=\frac{1}{N_{\mathrm{high}}}\sum_{i\in I_{\mathrm{high}}}(E(i)) \end{array}$$

where E(i) is the expression level of genes in cell i, $I_{\mathrm{low}}$, and $I_{\mathrm{high}}$ are the sets of cells with $t_{\mathrm{ANN}}<0.2$ and $t_{\mathrm{ANN}}>0.8$, respectively, and $N_{\mathrm{low}}$ and $N_{\mathrm{high}}$ are the number of cells in these sets.

The directionality of the ANN predictions was determined by comparing $\mathrm{Cytotrac}e_{\mathrm{low}}$ and $\mathrm{Cytotrac}e_{\mathrm{high}}$. If $\mathrm{Cytotrac}e_{\mathrm{high}}>\mathrm{Cytotrac}e_{\mathrm{low}}$, the latent time was assigned as $t_{p}=t_{\mathrm{ANN}}$; otherwise, it was assigned as $t_{p}=1-t_{\mathrm{ANN}}$.

Subsequently, we applied unsupervised clustering to categorize the cells. Clusters characterized by lower ANN times were designated as developmental origin states, while those with higher ANN times were identified as developmental end states:

$$\begin{array}{l} \mathrm{Developmental}\;\mathrm{Origin}\;\mathrm{States}\;\\ =\;\{\mathrm{clusters}\;\mathrm{with}\;\mathrm{low}\;t_{p}\;(\mathrm{first}\;10\%\;\mathrm{of}\;\mathrm{cells})\}\\ \mathrm{Developmental}\;\mathrm{End}\;\mathrm{States}\;\\ =\;\{\mathrm{clusters}\;\mathrm{with}\;\mathrm{high}\;t_{p}\;(\mathrm{last}\;10\%\;\mathrm{of}\;\mathrm{cells})\} \end{array}$$

To pinpoint the developmental intermediate states, we computed the DPT using a diffusion graph approach. The initial root node was selected based on clusters with the earliest mean latent time. The DPT, $t_{\mathrm{DPT}}$ was calculated, and clusters corresponding to the central portion of the pseudotime distribution were defined as developmental middle states:

$$\mathrm{Developmental}\;\mathrm{Middle}\;\mathrm{States}\;=\;\{\mathrm{clusters}\;\mathrm{with}\;t_{\mathrm{DPT}}\;\mathrm{around}\;\mathrm{the}\;\mathrm{median}\;(10\%\;\mathrm{cells})\}$$

This integrative approach, using both ANN time and DPT, allows for a nuanced understanding of the cellular differentiation trajectory, highlighting key transitional states from origin to endpoint.

### 2.6 Position fluctuation threshold

The Pre-ANN model consists of four linear layers as follows:

- fc1: This layer maps input features to a 512-dimensional hidden representation.
- fc2 and fc3: Both layers maintain a dimensionality of 512 and are followed by a ReLU activation function to introduce non-linearity.
- fc4: The final layer reduces the dimensionality from 512 to 1, producing a single output value.

Then, from the penultimate layer, extract the 512-dimensional vector v(L−1), which serves as the embedding for each cell. And Construct the neighborhood graph G using “scanpy.pp.neighbors” with v(L−1) as input. This function calculates the local neighborhood of each cell in the high-dimensional space defined by the embeddings. Besides, perform clustering on the neighborhood graph G using the Leiden algorithm “scanpy.tl.leiden” to identify distinct cell clusters C. All arguments set as default.

To calculate the position fluctuation threshold, we first sorted the identified cell clusters C based on their Pre-ANN time, which is a temporal attribute associated with each cluster. Compute the differences between adjacent Pre-ANN times:

$$\Delta t_{i}=t_{i+1}-t_{i}$$

Calculate the average difference $\overset{↼}{\Delta }t$ to determine the position fluctuation threshold for identifying origin and terminal cells:

$$\bar{\Delta t}=\frac{1}{n-1}\sum_{i=1}^{n-1}\;\Delta t_{i}$$

where: X: Pre-ANN model., *L*: number of layers in the Pre-ANN model., $v^{\left(L-1\right)}:$ vector from the penultimate layer of the Pre-ANN model., G: neighborhood graph., C: cell clusters. $t_{i}$: Pre-ANN time of the ith cell cluster. $\Delta t_{i}$: difference in Pre-ANN time between adjacent cell clusters. $\overset{↼}{\Delta }t$: average difference in Pre-ANN time. n: total number of cell clusters.

### 2.7 Latent time neural network framework

We assumed that the DPT followed the double weibull distribution (X∼dweibull(k,μ,σ)) (Balakrishnan and Kocherlakota 1985), and the LSI indexed neural network time adhered to the normal distribution ($X∼N(\mu ,\sigma^{2})$) (Ahsanullah *et al.* 2014). The latent time neural network (LTNN) was constructed using these two distributions:

LTNN=fdweibullfdweibull+fnorm×tLSI-Re-ANN+fnormfdweibull+fnorm×tdpt_pseudotimefdweibull=k2(x-μσ)k−1e-(x-μσ)kfnorm=1σ2πe-(x-μ)22σ2

Here, tLSI-Re-ANN represents the pseudotime calculated by Re-ANN model, $t_{\mathrm{dpt}\_\mathrm{pseudotime}}$ represents the psudotime calculated by diffusion graph implemented in partition-based graph abstraction (PAGA) methods. The SciPy library in Python (Virtanen *et al.* 2020) was utilized to estimate the parameters *k*, μ, and σ of the dweibull and normal distributions using maximum likelihood estimation.

### 2.8 Model evaluation metrics

Pre-ANN model’s performance was assessed using metrics such as accuracy, loss, and MAE. During the training phase, we utilized an 80–20 train-test split to evaluate the model’s generalization capability. The loss was measured using the mean squared error (MSE) criterion, which quantifies the average of the squares of the errors between predicted and true pseudotime values. The MAE was also calculated to provide an intuitive measure of prediction accuracy by averaging the absolute differences between predicted and true values.

### 2.9 Determining the PAGA-graph in scLTNN

PAGA graph abstraction has benchmarked as top-performing method for trajectory inference. It provides a graph-like map of the data topology with weighted edges corresponding to the connectivity between two clusters. Here, PAGA was extended in the scLTNN model by pseudotime-inferred directionality. We used LTNN-time as prior time key to calculate the transitions confidence.

In PAGA graph, pseudotime values are denoted by t. For each cell i, edges are retained only if the pseudotime of the target cell j satisfies:

$$t_{i}<t_{j}\;\mathrm{and}\;\underline{t_{j}}>\;\underline{t_{i}}-\epsilon$$

where $\underline{t_{i}}$ and $\underline{t_{j}}$ are the mean pseudotime values of the clusters containing cells i and j, respectively, and ϵ=0.1.

We then need to calculate the transition confidence matrix T,

$$T_{ij}=\frac{w_{ij}}{\sqrt{N_{i}\cdot N_{j}}}$$

where $w_{ij}$ is the transition weight between clusters i and j, and $N_{i}$ and $N_{j}$ are the sizes of clusters i and j, respectively. A threshold is applied to retain only the most confident transitions, this threshold is defined as:

$$\mathrm{threshold}=\max(\min(\max(\frac{T}{T>0},\mathrm{axis}=0))-10^{-6},\;0.01)$$

This ensures that only transitions exceeding the minimum of the maximum normalized transition weights are considered. To simplify the graph, a minimum spanning tree (MST) is optionally computed. If an MST is desired, indirect paths with higher confidence are retained by comparing direct transition weights with the maximum of indirect path weights.

### 2.10 Comparing the existing trajectory inference methods

We evaluated seven established trajectory inference algorithms—VIA, Palantir, Slingshot, scVelo, VeloVI, DPT, and SCENT—employing scvelo.tl.paga across all methods to uniformly compute cell type transition confidences. Detailed descriptions of individual algorithmic implementations, along with parameter settings, are documented in our GitHub repository. For human bone marrow hematopoietic cell differentiation, we manually annotated eight transformation confidence nodes, denoted as: HSC_1→HSC_2, HSC_2→Precursors, Precursors→Mono_1, Precursors→Mono_2, Mono_2→DCs, HSC_1→Ery_1, Ery_1→Ery_2, and HSC_1→Mega. Similarly, for mouse pancreatic cell differentiation, we annotated six nodes, as follows: Ngn3 low EP→Ngn3 high EP→Pre-endocrine, Pre-endocrine→Alpha, Pre-endocrine→Beta, Pre-endocrine→Delta, and Pre-endocrine→Epsilon.

Transitions confidence and Probability of Transitions rate: The transitions probability is an evaluation metric that first appeared in scVelo, we optimized it to “probability of transitions rate” as a probability metric for state transitions. Simultaneously, the confidence of the transitions probability, namely “transitions confidence” was also computed using “scvelo.tl.paga.” We defined the transformation success probability as a non-zero confidence value.

Terminal rate prob: Regarding the terminal cell transition ratio, we classified cells with pseudotime values exceeding 0.95 as “temporal terminal cells” and then determined the ratio of these cells to “true terminal cells” to assess the biological accuracy of the algorithms.

Hamming distance: To compare the similarity of cell development trajectory networks, we applied the HIM metric (Jurman *et al.* 2015), which inputs two weighted adjacency matrices of the milestone networks, weighted by edge length. This metric is a linear composition of the normalized Hamming distance and the normalized Ipsen–Mikhailov distance, reflecting edge length differentials and degree distribution similarities, respectively. The latter incorporates a fixed parameter *γ*, set at 0.1 to ensure comparability across datasets. The Hamming distance can be calculated via netrd.distance.Hamming, accessible at https://netrd.readthedocs.io/en/latest/distance.html.

Jaccard distance: In assessing cell development trajectory network branch assignments, we utilized the F1 score to gauge branch assignment accordance, similar to approaches used in comparing biclustering methods (Saelens *et al.* 2018). Initial assessment entailed calculating paired branch similarities using the Jaccard index, subsequently computing the Jaccard distance through netrd.distance.JaccardDistance.

## 3 Results

### 3.1 The distribution of highly variable gene expression across multiple tissues/organs in both human and mouse exhibits a high degree of similarity

**Figure 2.**
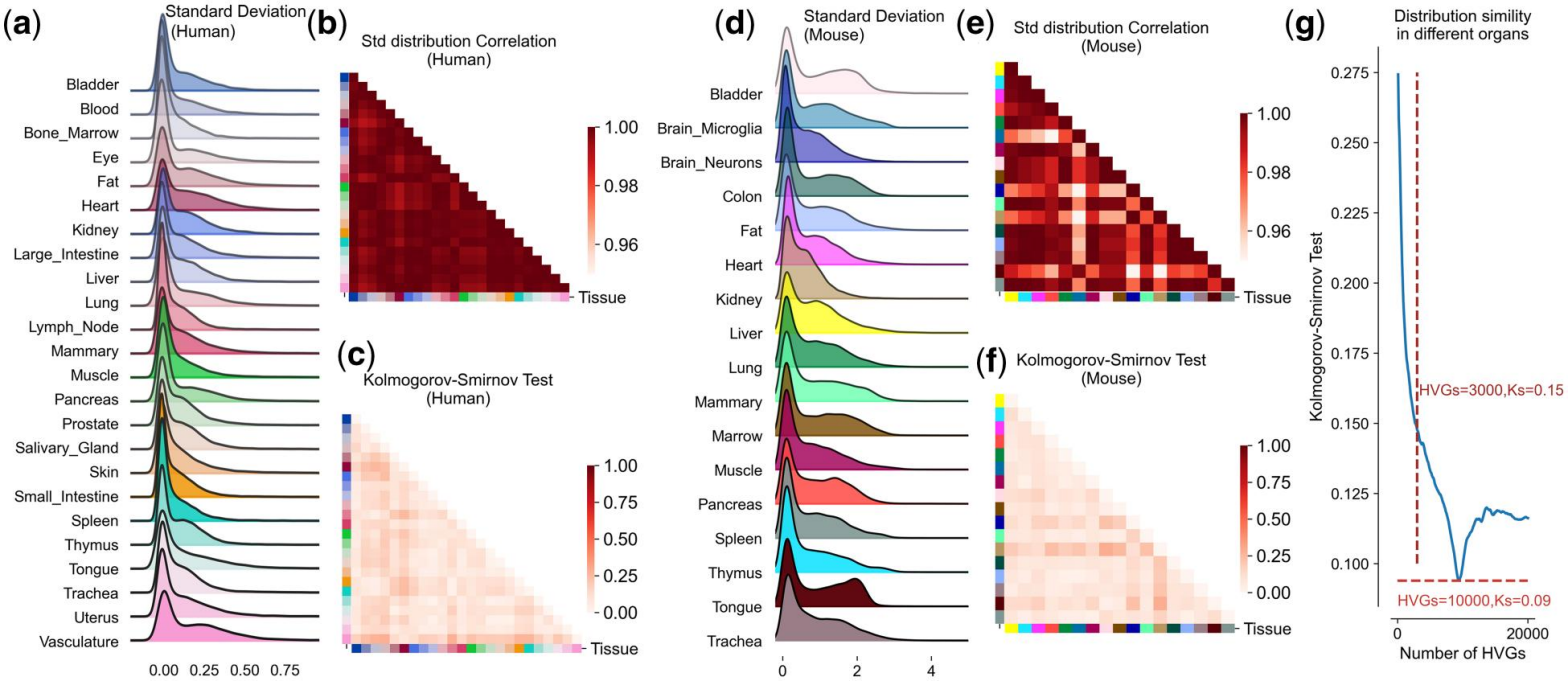
Distribution regularity of HVG expression across multiple tissues and organs in human and mouse. (a) The standard deviation (Std) of the expression of the top 10 000 HVGs across 24 human tissues and organs. (b) The distribution correlation of Std values in cells of 24 human tissues and organs. (c) The Kolmogorov-Smirnov Test values in cells of 24 human tissues and organs. (d) The standard deviation (Std) of the expression of the top 10 000 HVGs across 17 mouse tissues and organs. (e) The distribution correlation of Std values in cells of 17 mouse tissues and organs. (f) The Kolmogorov-Smirnov Test values in cells of 17 human tissues and organs. (g) The distribution of Std simility in different number of HVGs. Std, standard deviation.

To construct the Pre-ANN model, the first step involved examining the HVGs. HVGs are commonly used in characterizing cell subpopulations and identifying specific marker genes for distinct cell types (Stegle *et al.* 2015). However, the regularity on distribution and characteristics of gene expression levels for HVGs across different organs or tissues remains elusive. We used Scanpy to identify the top 10 000 HVGs on scRNA-seq data from 24 human tissues and organs (Liu and Zhang 2022). These genes were subsequently analyzed using a ridge plot (Fig. 2a). Intriguingly, the distribution patterns of the standard deviation (Std) were remarkably consistent across all 24 human tissues/organs and 17 mouse tissues/organs (Fig. 2a and d). Furthermore, the correlation coefficients of these values among the 24 human tissues/organs and 17 mouse tissues/organs consistently exceeded 0.98 (Fig. 2b and e). To compare the consistency of the distribution across different tissues/organs, we evaluated the similarity of the distribution among various organs using the Kolmogorov-Smirnov test. In the case of 24 human tissues/organs, the average Kolmogorov-Smirnov test score was less than 0.2 (Fig. 2c), and similarly, in 17 mouse tissues/organs, the average score was also less than 0.2 (Fig. 2f). These results suggest a high consistency in feature distribution among organs, as derived from 10 000 HVGs. We will refer to the HVGs of different organs as meta-HVGs.

To clarify the impact of the number of HVGs on the distribution of organ features, we examined the inter-organ feature scores derived from 100 to 20 000 HVGs. At 3000 HVGs, the slope of the Kolmogorov-Smirnov test score undergoes a sudden change and begins to be less than 0.15. Furthermore, at 10 000 HVGs, the Kolmogorov-Smirnov test score reaches a minimum value of 0.09 (Fig. 2g and Supplementary Fig. S1a and b). The specific gene names of these meta-HVGs may vary, but the first 10 000 HVGs of each organ represent the unique characteristics of that organ.

In addition to this, we also evaluated the similarity of organ distribution in scRNA-seq datasets derived from different library constructs. The Kolmogorov-Smirnov test score for the distribution of the standard deviation of the first 10 000 HVGs from different organs obtained by the Smart-seq technique was 0.077, while that of the 10× genomic technique was 0.13 (Supplementary Fig. S1c and d). This suggests that despite a significant difference in organ distribution between different sequencing platforms, the difference in the distribution of meta-HVGs between organs of sequencing libraries constructed by the same technology was minimal. These findings present an intriguing viewpoint: even though the specific genes with the highest variability may differ among single-cell data from different tissues, organs, or sources, the distribution characteristics of the most variant genes exhibit remarkable similarity. This suggests a common underlying pattern in the gene expression profiles of HVGs across a variety of biological contexts.

### 3.2 Constructing scLTNN model based on Pre-ANN and Re-ANN models

Given the observed feature similarity of HVGs, we postulated that a Pre-ANN model, built using HVGs from a single cell atlas of one organ, could potentially generalize well to other single-cell atlases within the same species. To put this hypothesis to the test, we initially constructed the Pre-ANN model using human pancreatic epithelial cells (hPECs) (Fig. 1a). We extracted the top 10 000 variable genes of hPECs, and their features were captured and labeled using the LSI, an indexing method that reflects gene knowledge and relationships, akin to Natural Language Processing (NLP) (Li *et al.* 2022). The cells labeled with latent time by the LSI were then subjected to dimensionality reduction, resulting in a latent semantic vector. This vector was used as input for the Pre-ANN regression model, thus constructing a primary Pre-ANN regression model. Concurrently, the latent times of each cell were calculated by VeloVI based on RNA velocity, resulting in the latent time vector. Ultimately, this latent time vector was used for training the Pre-ANN regression model with a regression loss function, as depicted in the lower panel of Fig. 1a.

**Figure 3.**
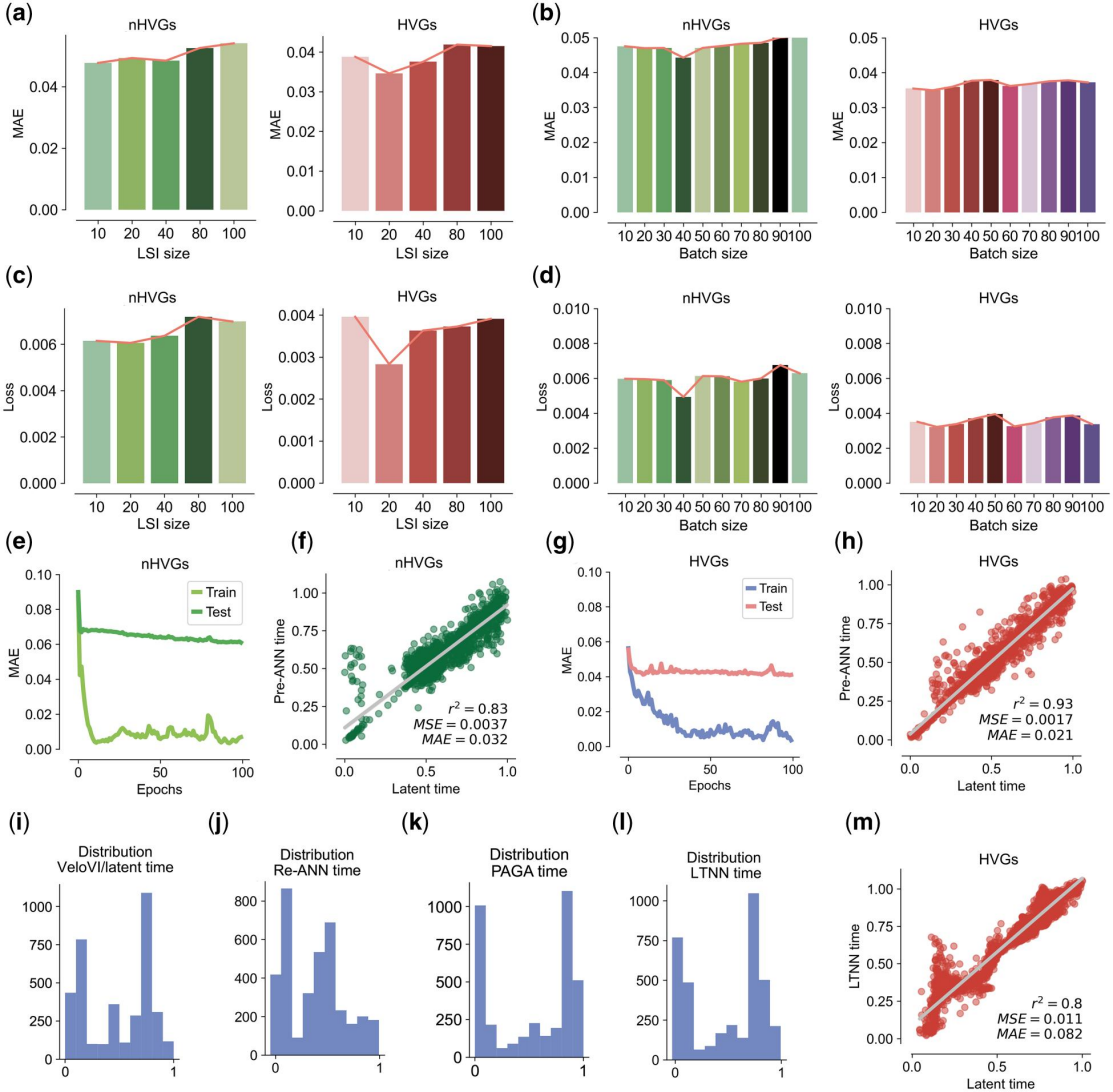
Model training of Pre-ANN and Re-ANN. (a–d) Optimization of the sizes of LSI and batch. (a) The MAE of distinct LSI sizes. (b) The MAE of distinct batch sizes. (c) The loss of distinct LSI sizes. (d) The loss of distinct batch sizes. (Left: nHVGs; Right: HVGs). (e) The MAE dramatically decreased after Pre-ANN training using nHVGs. (f) The regression of Pre-ANN time (predicted by Pre-ANN model using nHVGs) and latent time by VeloVI. (g) The MAE dramatically decreased after Pre-ANN training using HVGs. (h) The regression of Pre-ANN time (predicted by Pre-ANN model using HVGs) versus latent time (predicted by VeloVI). The distribution of latent time of the pancreas cells calculated by VeloVI (i), Re-ANN (j), PAGA (k), and LTNN (l). (m) The regression of scLTNN time versus VeloVI latent time. HVGs, high-variable genes; nHVGs, non-high-variable genes; MAE, mean absolute error; MSE, mean squared error; LTNN, latent time neural network.

During the construction of the Pre-ANN regression model, we employed MAE (as shown in Fig. 3a and b) and loss (as depicted in Fig. 3c and d) to fine-tune the LSI sizes and batch sizes for the model’s prediction regression effect evaluation. The model, when trained on the HVGs, achieved its lowest MAE when both the LSI and batch size were set to 20 (as seen in Fig. 3a and b). This finding was in line with the results from the loss metric analyses (Fig. 3c and d). Therefore, the LSI and batch sizes of 20 were determined as the optimal settings for training the Pre-ANN regression model. When compared to the MAE of the model trained on nHVGs, the MAEs of the model trained on HVGs were 10 times lower (Fig. 3e–h). The Pre-ANN times, as determined by the Pre-ANN model, closely matched the latent times calculated by VeloVI (Fig. 3h).

Next, the Pre-ANN time were generated by Pre-ANN model using the LSI matrix of the previously processed HVGs from single-cell matrix as input. The cells that remained in the top 5% and bottom 5% of the Pre-ANN time were annotated as the origin and end cell stages, respectively. In conjunction with CytoTRACE, a method based on transcriptional diversity changes during cellular differentiation—the number of genes expressed in a cell decreases during differentiation (Gulati *et al.* 2020), we indirectly eliminated the dependency on the specific values of Pre-ANN time using position fluctuation threshold and determined the origin and end states (upper panel of Fig. 1b). At the same time, the cells that remained in the middle spectrum of Pre-ANN time were extracted, and the DPT was determined using partition-based graph abstraction (Wolf *et al.* 2019). The cell cluster that remained in the median of DPT_PAGA time was set as the middle cells of the trajectory (lower panel of Fig. 1b).

To enhance the precision of DPT prediction, we constructed the repeated artificial neural network (Re-ANN) regression model (as shown in the right panel of Fig. 1b). The Re-ANN model was trained using the LSI values of the origin, middle, and end cells mentioned earlier as input, and the normally distributed latent times of the origin, middle, and end cells [we assigned *N*∼(0.05, 0.1) to origin cells, *N*∼(0.5, 0.1) to middle cells, and *N*∼(0.95, 0.1) to end cells, seen details in Methods 2.5] as the output (as shown in the right panel of Fig. 1b). Subsequently, the Re-ANN time of all cells, including those not included in the origin, middle, and end cells, was obtained by inputting their LSI values into the Re-ANN model.

Compared to the latent times of VeloVI, which show a canonical distribution with a three-peaked pattern of high-low-high-low-high, and the DPTs of PAGA, which satisfy a double Weibull distribution, the Re-ANN pseudotimes exhibited a normal distribution. This suggests a potential deviation in accurately reflecting the origin and end states of the cells by Re-ANN (as shown in Fig. 3i–k). By integrating the pseudotime distribution of Re-ANN and PAGA, we were able to create a latent time neural network model termed LTNN. The pseudotimes predicted by this model show similar distribution characteristics to those of VeloVI and PAGA (as shown in Fig. 3l). When we regressed LTNN time against VeloVI latent time, we found a proportionate fit (as shown in Fig. 3m).

### 3.3 Predicting the origin and end cells by scLTNN in different species

**Figure 4.**
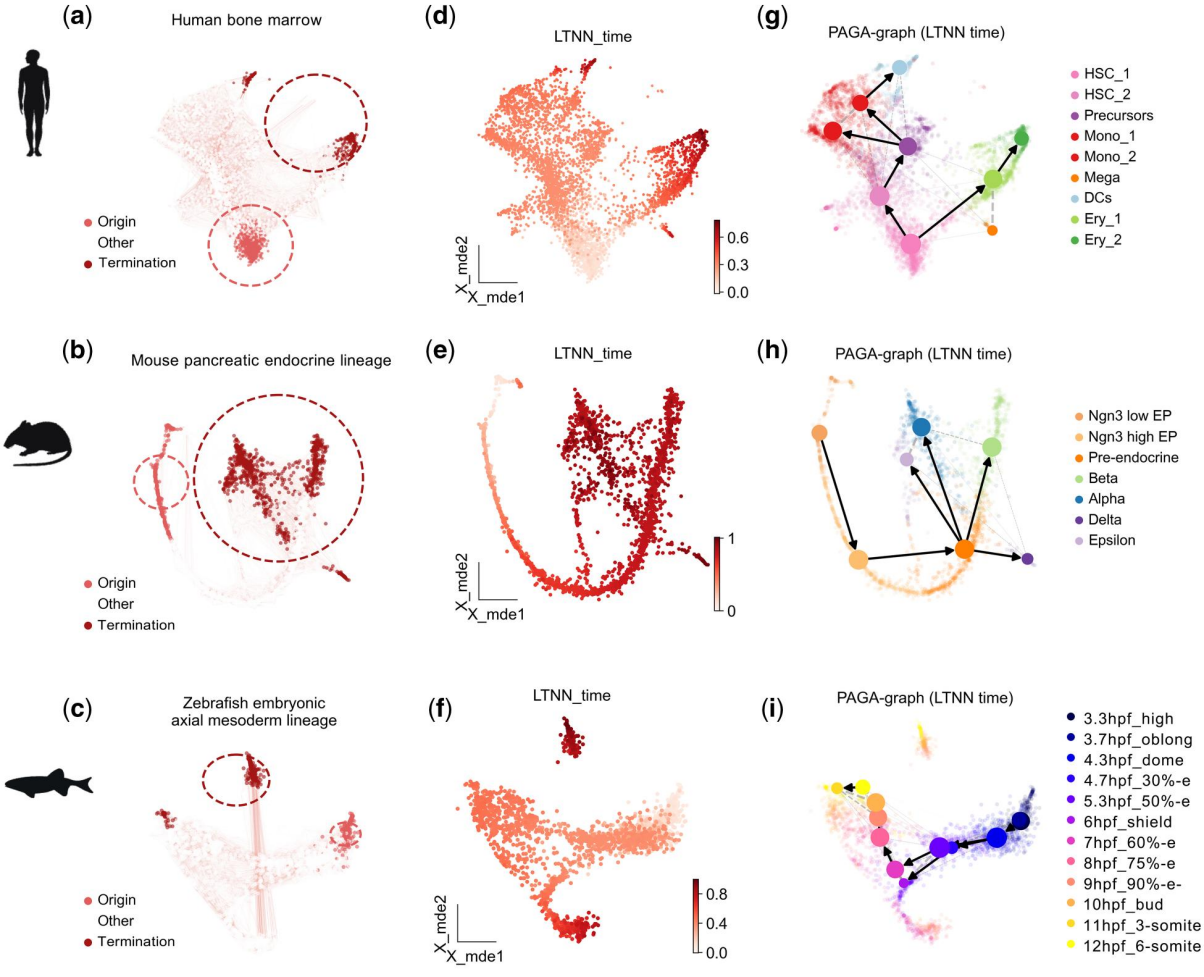
Single-cell trajectory of human bone marrow cells, mouse pancreatic endocrine lineage, and axial mesoderm lineage of zebrafish embryo depicted by scLTNN. (a–c) UMAP displaying the origin and end cells in human bone marrow cells (a), mouse pancreatic endocrine lineage (b), and axial mesoderm lineage of the zebrafish embryo (3.3–12 h) (c). (d–f) UMAP displaying the LTNN times of human bone marrow cells (d), mouse pancreatic endocrine lineage (e), and axial mesoderm lineage of the zebrafish embryo (3.3–12 h) (f). (g–i) Transition confidence using the PAGA-graph module of scLTNN model in human bone marrow cells (g), mouse pancreatic endocrine lineage (h), and axial mesoderm lineage of the zebrafish embryo from 3.3hpf to12hpf (i).

We then proceeded to test the applicability in inferring cell trajectories of pre-trained scLTNN for human, mouse, and zebrafish, including human bone marrow, mouse pancreatic endocrine lineage with Ngn3-Venus fusion cells during the secondary transition, and zebrafish embryonic axial mesoderm lineage ranging from 3.3 to 12 hours post fertilization (hpf). The scLTNN was able to efficiently identify the origin and end cells in each of the three cell clusters from human, mouse, and zebrafish tissues, respectively (Fig. 4a–c and Supplementary Fig. S2). For the human bone marrow cells, the scLTNN identified hematopoietic stem cells (HSCs) as the origin cells and erythrocytes as the end cells (Supplementary Figs S2a–c and S3b). In the case of mouse pancreatic endocrine lineage, the endocrine progenitor Ngn3 low EP were marked as the origin state and the Beta cells as the end state (Supplementary Figs S2d, f, and S3d). For zebrafish embryonic cells, cells from the 3.3hpf_high stage were identified as the origin cells, while the 12hpf_6-somite cells were marked as the end cells (Supplementary Figs S2g–i and S3f). These findings accurately reflect the developmental stages of these cells (Farrell *et al.* 2018, Bastidas-Ponce *et al.* 2019, Setty *et al.* 2019), demonstrating the robustness and precision of the scLTNN model in identifying the origin and end cells across different species and various biological contexts.

Based on the pseudotimes assigned to each cell by scLTNN, we were able to generate the developmental trajectories of human bone marrow cells, mouse pancreatic endocrine lineage, and zebrafish embryonic axial mesoderm lineage cells (Fig. 4d–i and Supplementary Fig. S3). We also used the conventional DPT method for parallel comparison (Supplementary Fig. S3). Both PAGA and scLTNN performed similarly in depicting the developmental trajectories of mouse pancreatic cells and zebrafish embryonic cells, successfully obtaining the developmental trajectories that accurately mirror the physiological development of these cells (Fig. 4e–i and Supplementary Fig. S3c–f) (Farrell *et al.* 2018, Bastidas-Ponce *et al.* 2019, Setty *et al.* 2019). However, for the human bone marrow cells, DPT defined megakaryocytes as differentiated from erythroid cells [seen “PAGA-graph (DPT)” in Supplementary Fig. S4], suggesting that DPT was unable to correctly assign megakaryocytes to the appropriate developmental stages of bone marrow cells. Although the scLTNN did not identify megakaryocytes as terminal cells (Fig. 4g and Supplementary Figs S3a, b, and S4 “PAGA-graph (LTNN)”], but avoided erroneous “erythroid cells-megakaryocytes” trajectory identification.

### 3.4 scLTNN demonstrates superior performance in trajectory inference

**Figure 5.**
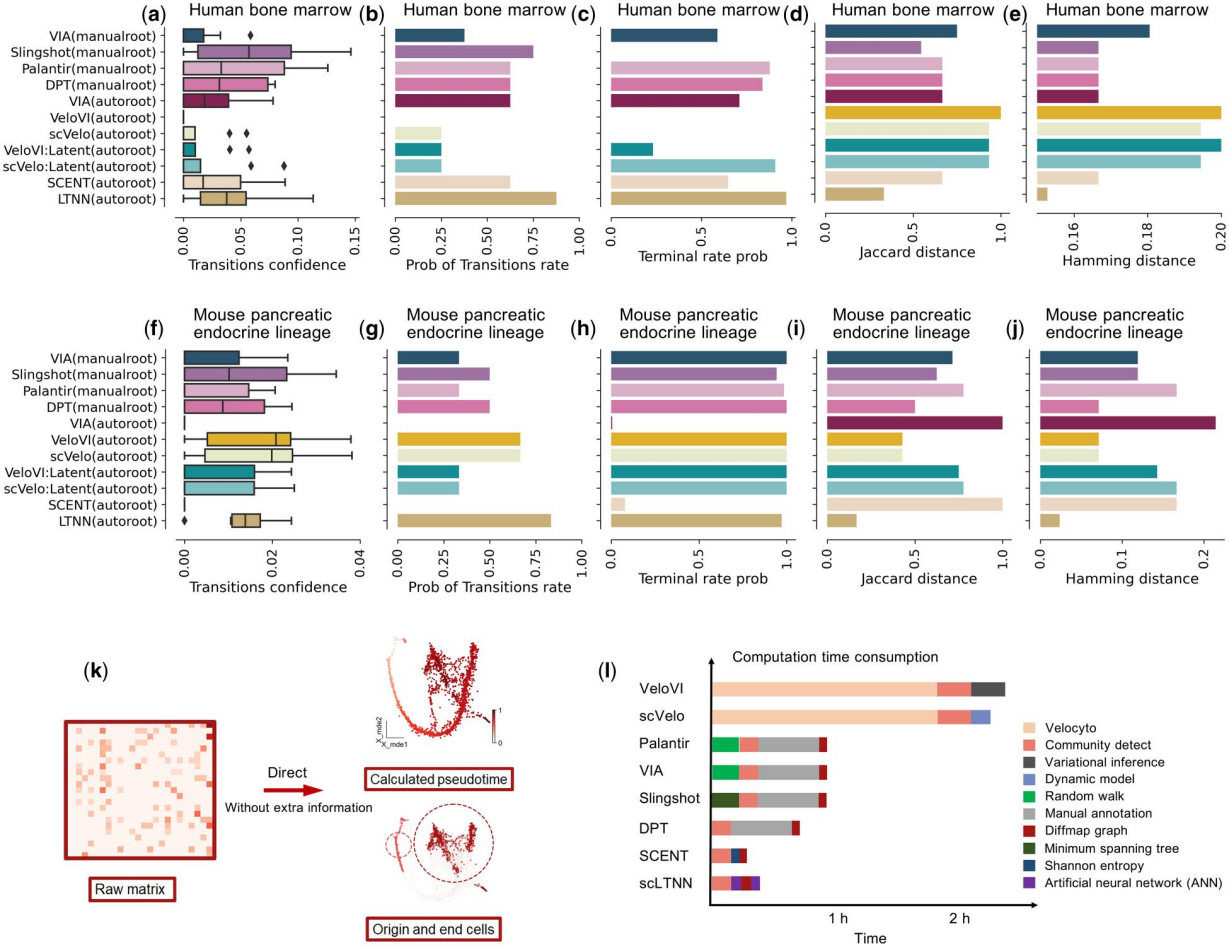
scLTNN exhibits a superior performance in trajectory inference. (a–e) Evaluating the performance of the indicated methods in inferring the trajectory of human hematopoietic cell differentiation, which includes the following pathways: HSC_1->HSC_2->Precursors->Mono_1->Mono_2->DCs, HSC_1->Ery_1->Ery_2, and HSC_1->Mega. (a) The confidence level of indicated methods for cell type transformation during human bone marrow hematopoietic differentiation is variable. If the transformation fails, the confidence level is set to 0, indicating no assurance of successful differentiation. (b) The rate of successful cell transformation is calculated from the percentage of transformation confidence nodes that are not 0 in the overall transformation confidence nodes (see details in Section 2). (c) The terminal cell prediction probability refers to the likelihood of a terminal cell appearing in the proposed chronology after 95% of the proposed chronology has been calculated. This probability provides an estimate of the potential for a terminal cell to occur within the remaining 5% of the chronology. (d) The Jaccard distance, calculated as one minus the size of the intersection of the sets divided by the size of their union, reflecting the distance from the real transformation. (e) The Hamming distance measures the difference between two sequences of equal length by counting the number of positions at which the corresponding elements are different, also reflecting the distance from the real transformation. (f–j) Evaluating the performance of the indicated methods in inferring the trajectory of mouse pancreatic endocrine lineage, which includes the following pathways: Ngn3 low EP-> Ngn3 high EP-> Pre-endocrine->Alpha/Beta/Delta/Epsilon. The analysis of the transition confidence (f), the transition rate (g), the terminal cell prediction probability (h), the Jaccard distance (i), and the Hamming distance (j) were similar to those in (a–e). (k) Sketch map of pseudotime inference. (l) Computation time consumed by distinct pseudotime inference methods. Although velocyto-dependent models (e.g. VeloVI, scVelo) do not need prior knowledge of the biological process for manual annotation of the origin and end markers, they do need Velocyto, the computationally intensive process of calculating the unspliced/spliced matrices from the bam file, usually taking anywhere 1–2 h; While other algorithms require the manual annotation, which may need literature survey and cost more time.

In order to demonstrate that scLTNN can accurately infer biologically significant cell developmental trajectories, we selected human bone marrow hematopoietic cell differentiation and mouse pancreatic endocrine lineage as the ground truth, respectively, and uniformly compared a total of seven proposed time-series computational algorithms-VIA, Palantir, Slingshot, scVelo, VeloVI, DPT, and SCENT-to calculate the confidence of cell type transformation and the distance of the model calculation from real transformation using Jaccard distance and Hamming distance (Fig. 5a–j), which were widely applied to assess cell development trajectories (Jia and Chen 2022, Sumanaweera *et al.* 2025).

In the process of human bone marrow hematopoietic cell differentiation, we establish the ground truth as follows: HSC_1->HSC_2->Precursors->Mono_1/Mono_2->DCs, HSC_1->Ery_1->Ery_2, HSC_1->Mega, amounting to three differentiation pathways in total. We utilized four algorithms—VIA, Slingshot, Palantir, and DPT—with the developmental starting point manually selected, and another five algorithms—VIA, VeloVI, scVelo, SCENT, and LTNN—with the developmental starting point automatically calculated. Notably, VeloVI and scVelo computed both velocity-based pseudotime (VeloVI/scVelo) and dynamic model-based pseudotime (VeloVI/scVelo: Latent). Our findings indicate that scLTNN exhibits similar cell type transformation confidence to Slingshot and Palantir, but with superior robustness and 100% confidence in the proportion of states accurately identified (Fig. 5a and b). Regarding the proportion of terminal cells in cells with proposed timing values exceeding 0.95, scLTNN shares the same proportion with Palantir, DPT, and scVelo, all of which are greater than 95% (Fig. 5c). Furthermore, scLTNN consistently achieves the closest distance to the ground truth when compared with all other evaluated temporal algorithms (Fig. 5d and e).

In the process of mouse pancreatic endocrine lineage differentiation, we defined the differentiation pathways as Ngn3 low EP-> Ngn3 high EP-> Pre-endocrine-> Alpha/Beta/Delta/Epsilon, totaling four pathways. We discovered that scLTNN shares similar cell type transformation confidence with Slingshot, DPT, scVelo, and VeloVI (Fig. 5f). However, scLTNN exhibits superior robustness and maintains a 100% confidence ratio in accurately identifying states, while scVelo and VeloVI only achieve 80% (Fig. 5g). We also calculated the proportion of terminal cells in cells with proposed timing values greater than 0.95. scLTNN shares the same proportion with all other proposed timing algorithms, all exceeding 0.95 (Fig. 5h). Similarly, the values of Jaccard distance and Hamming distance of scLTNN were lowest among all the time-series computational algorithms (Fig. 5i and j), suggesting the robustness and accuracy of scLTNN consistently outperforming comparative algorithms.

## 4 Discussion

Compared to existing computational tools shown above, the novel tool scLTNN has indeed shown superior capabilities in automatically identifying the origin and end cells based on distributional similarity of multi-organ meta-HVGs, as well as calculating the pseudotime with higher accuracy of the proposed temporal order calculation, without the need for prior biological knowledge (Figs 4 and 5a–j and Supplementary Fig. S2). In addition, notably, scLTNN consumes less computation time than the aforementioned tools. Once *a priori* organ mapping of Pre-ANN has been pre-trained for the same species under the same sequencing platform, the scLTNN method requires significantly less computational time and resources while maintaining accuracy, compared to the more computationally intensive methods such as Velocyto or manual annotation (Fig. 5k–l).

The multi-organ single-cell sequencing data at the species level provides us with a powerful and robust map of prior knowledge (Schaum *et al.* 2018, Tabula Sapiens Consortium *et al.* 2022). By utilizing this prior knowledge, we can reduce the computational and time costs associated with predicting the origin and terminal cells. We generalize high-variability features from single-cell sequencing data of different organs of the same species, using the same sequencing platform, for neural network training. We discovered that a model trained with high-variability features calculated within one organ can be transferred to another organ for prediction. After correction using CytoTrace (Gulati *et al.* 2020), it can accurately identify the origin and terminal cell types. If batch effects need to be removed for pseudotime calculation, we can correct the matrix using CellANOVA (Zhang *et al.* 2024).scLTNN is the first trajectory inference model based on data distribution modeling, distinguishing itself from Palantir, which is based on Markov chains, and Diffusion Graph, which is based on graph coupling. scLTNN models the origin, middle, and terminal cell types with a pseudo-temporal normal distribution, and then uses neural networks to train the pseudo-time values of other cells. After training, we combine the double Weibull distribution to correct the pseudo-time values, presenting a tri-modal distribution. This is consistent with the good distribution obtained by Velocyto modeling latent time (Lopez *et al.* 2018, Bergen *et al.* 2020).

We incorporate the PAGA-graph into scLTNN for the computation of cell type state transition graphs (Wolf *et al.* 2019, Bergen *et al.* 2020). This model computes state transition graphs based on the cell neighbor graph and pseudo-time values, with the algorithm prototype implemented in VeloVI. The automatic recognition ability of scLTNN is comparable to the PAGA-graph calculated by manually labeling cell types based on pseudotime (Supplementary Figs S4 and S5). It achieves state-of-the-art results on multiple metrics, indicating that scLTNN can accurately capture the transition of cell type states.

However, it should be noted that scLTNN still has some limitations. For instance, the application of pre-trained scLTNN model is restricted in the same species. The pre-ANN model trained by human bone marrow dataset could correctly predict the start and end cells during human bladder mesenchymal cells to myofibroblasts (Supplementary Fig. S6a–c), but this generalization ability was suppressed in infer mouse bone marrow hematopoietic cell differentiation (Supplementary Fig. S6e–g). For single-cell sequencing data of an entirely new species, we lack pre-existing Pre-ANN models to use, which necessitates retraining. Furthermore, the similarity of features across multiple organs of the new species remains an unknown issue. The accuracy of model transfer has not been confirmed. Additionally, scLTNN is currently restricted to scRNA-seq data and cannot include cells not on the differentiation trajectory. This is a common issue among trajectory inference algorithms, and scLTNN has not proposed a new solution, nor is it suitable for spatial RNA-seq data.

In summary, scLTNN is a comprehensive and robust trajectory inference algorithm, including functions such as cell state identification, pseudo-temporal calculation, and construction of cell state transition graphs for trajectory inference. It achieves the effect of manual recognition in the task of automatically identifying trajectories, significantly reducing the time required for trajectory inference. It is available at https://github.com/Starlitnightly/scLTNN

## Supplementary Material

vbaf033_Supplementary_Data

## Data Availability

All the preprocessed data can be downloaded from Figshare (Zeng 2023). The scLTNN Python package, which is used for the scLTNN framework, along with the scripts for training and evaluation via the Jupyter notebook, are available at https://github.com/Starlitnightly/scltnn.
