# Supplementary material for: scLTNN: an innovative tool for automatically visualizing single-cell trajectories
Source: Bioinform Adv. 2025 Feb 26;5(1):vbaf033. doi: 10.1093/bioadv/vbaf033 (PMC11889453; doi:10.1093/bioadv/vbaf033)
Supplement: vbaf033_Supplementary_Data [file vbaf033_supplementary_data.docx]

# Supplementary figures


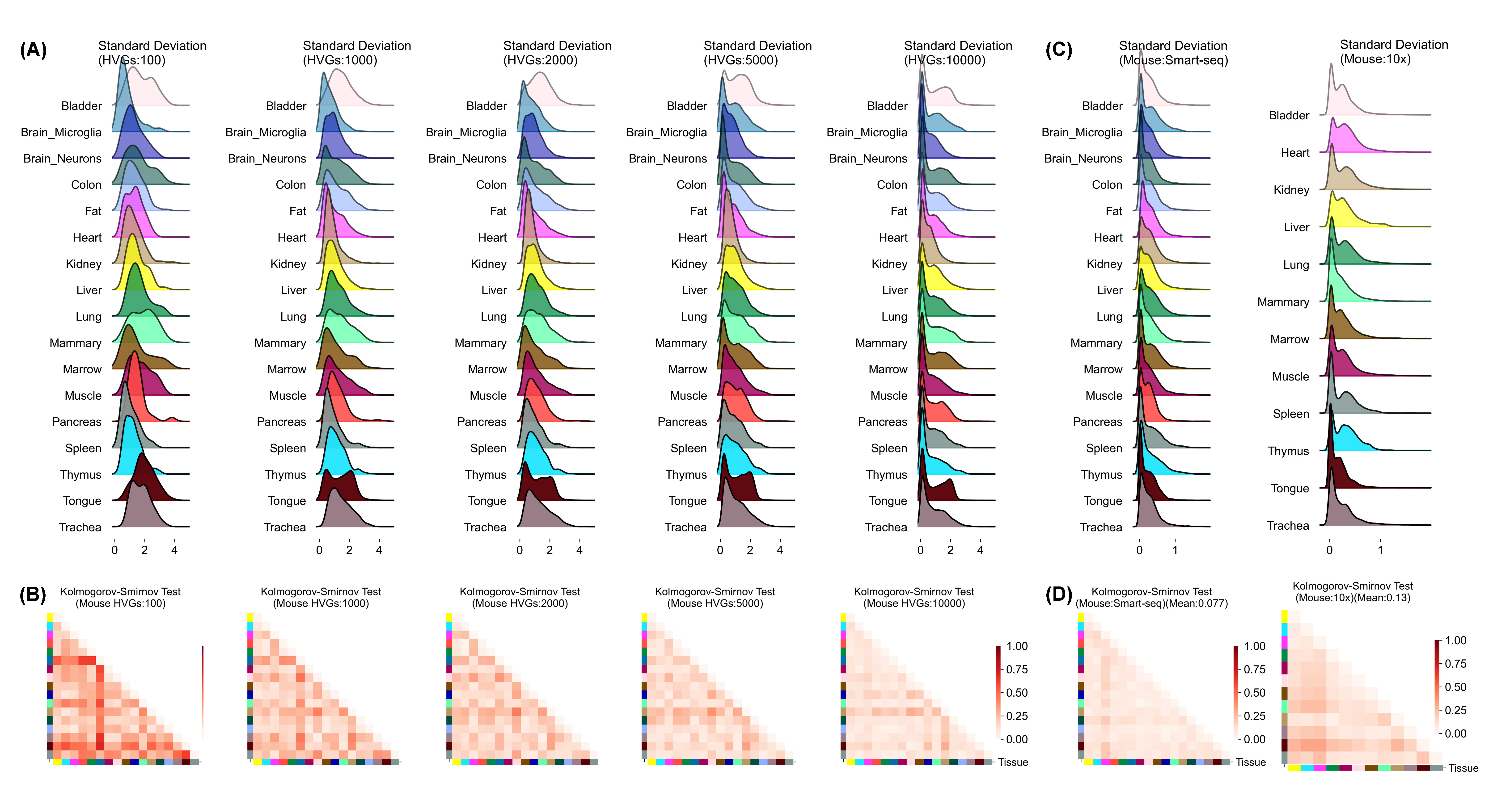


**Figure S1 Distribution regularity of HVG expression across mouse organs.** (A) The standard deviation (Std) of the expression of the top 100-10,000 HVGs across 17 mouse tissues and organs. (B) The Kolmogorov-Smirnov Test values of the top 100 to 10,000 HVGs in cells across 17 mouse tissues and organs. (C) The standard deviation (Std) of the expression of the top 100-10,000 HVGs across 17 mouse tissues/organs in smart-seq (left) and across 11 mouse tissues/organs in 10x genomics (right). (D) The Kolmogorov-Smirnov Test values of the top 100 to 10,000 HVGs in cells across 17 mouse tissues and organs in smart-seq (left) and 10x genomics (right).


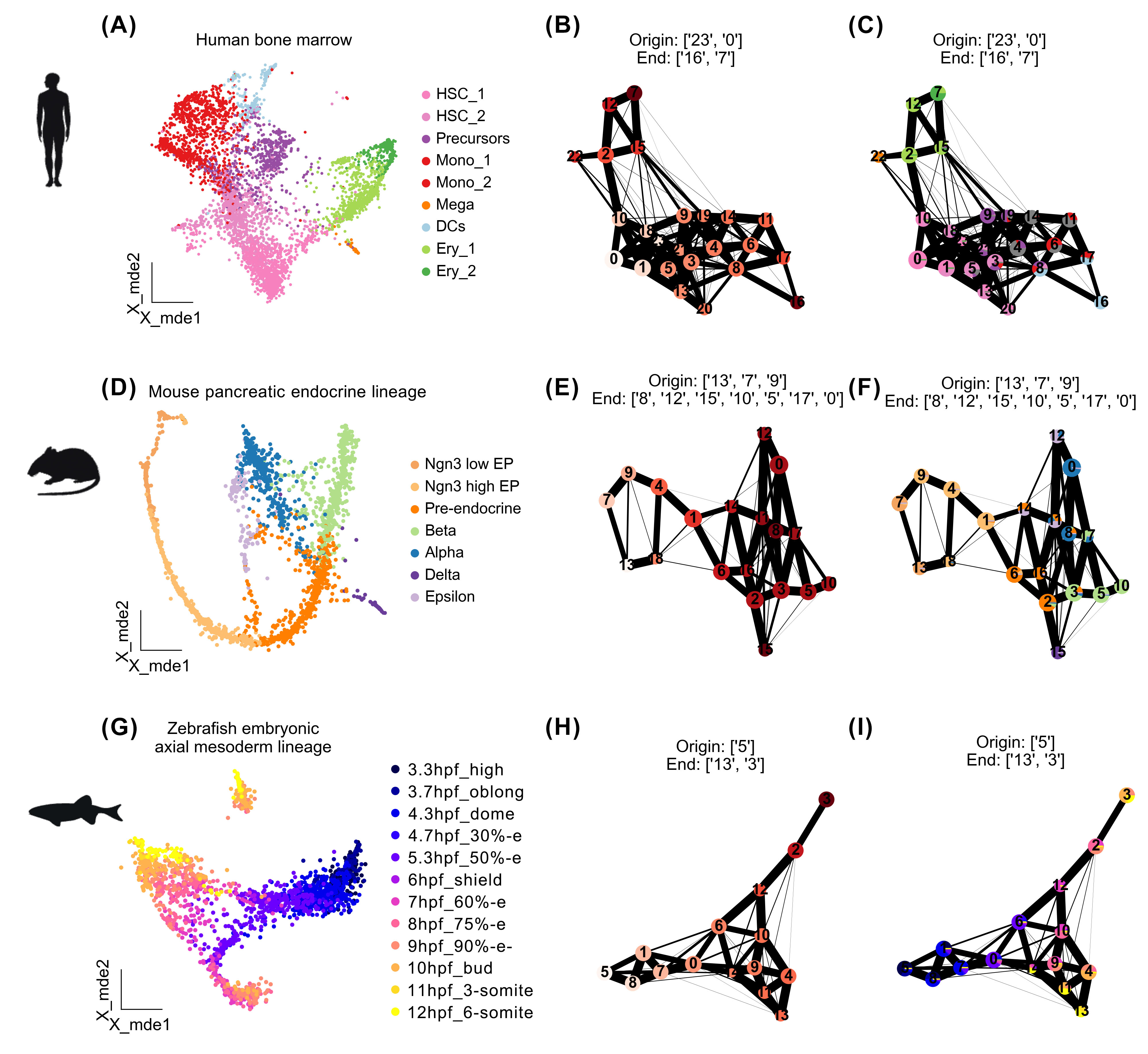


**Figure S2 Identifying the origin and end cells of human bone marrow cells, mouse pancreatic endocrine lineage, and axial mesoderm lineage of zebrafish embryo.** (A) UMAP showing the 9 human bone marrow cell subsets. (B) Scoring the state of human bone marrow cell subsets. The 9 human bone marrow cell subsets were divided into 23 cellular community (from 0 to 22) according to Re-ANN time. The cellular community ‘23, 0’ were defined as origin cells; the cellular community ‘16, 7’ was defined as end cells. (C) The cell composition of each cellular community of human bone marrow cells. (D) UMAP showing the 8 distinct mouse pancreatic endocrine lineage. (E) Scoring the state of mouse pancreatic endocrine lineage. The 8 mouse pancreatic endocrine lineage were divided into 19 cellular community (from 0 to 18) according to Re-ANN time. The cellular community ‘13, 7, 9’ were defined as origin cells; the cellular community ‘8, 12, 15, 10, 5, 17, 0’ were defined as end cells. (F) The cell composition of each cellular community of mouse pancreatic endocrine lineage. (G) UMAP showing the 12 distinct subsets of zebrafish embryo axial mesoderm lineage. (H) Scoring the state of subsets of zebrafish embryo axial mesoderm lineage. The 12 zebrafish embryo axial mesoderm lineage subsets were divided into 15 cellular community (from 0 to 14) according to Re-ANN time. The cellular community ‘5’ was defined as origin cells; the cellular community ‘13, 3’ was defined as end cells. (I) The cell composition of each cellular community of zebrafish embryo axial mesoderm lineage. HSC, hematopoietic stem cells; DC, dendritic cells; Ery, erythroid cells; Mega, megakaryocytes; Mono, monocytes; EP, early precursors; Alpha, glucagon-producing α-cells, Beta, insulin-producing β-cells; Delta, somatostatin-producing δ-cells; Epsilon, ghrelin-producing ε-cells.


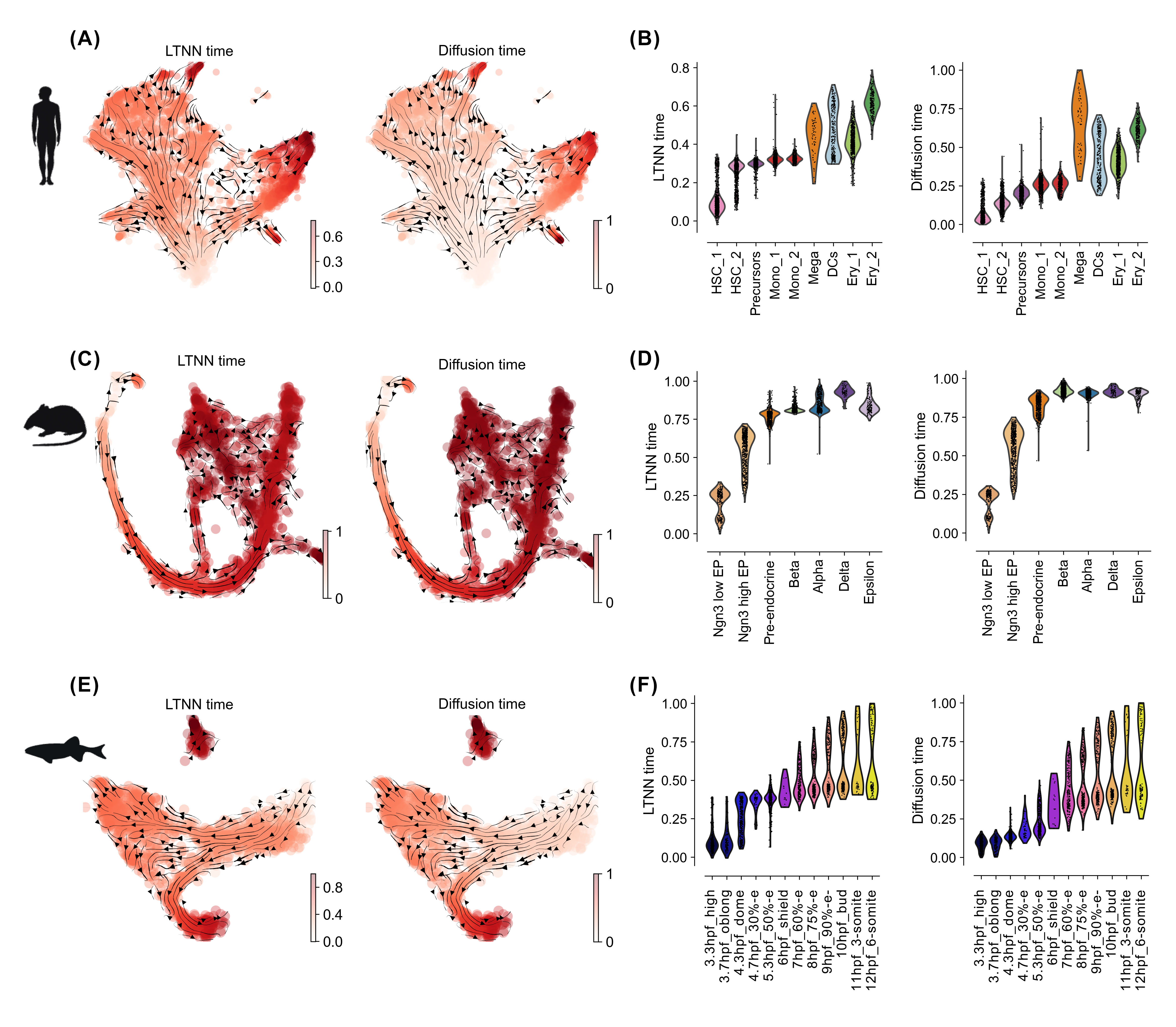


**Figure S3 Comparing the single-cell trajectories determined by LTNN and DPT.** (A) UMAP showing the single-cell trajectories of human bone marrow cells determined by LTNN (left) and DPT (right). (B) Violin plots showing the pseudotimes of each human bone marrow cell subsets determined by LTNN (left) and DPT (right). (C) UMAP showing the single-cell trajectories of mouse pancreatic endocrine cells determined by LTNN time (left) and DPT (right). (D) Violin plots showing the pseudotimes of each mouse pancreatic endocrine cell subsets determined by LTNN (left) and DPT (right). (E) UMAP showing the single-cell trajectories of the zebrafish embryo axial mesoderm lineage (3.3h-12h) determined by LTNN (left) and DPT (right). (F) Violin plots showing the pseudotimes of each subsets of zebrafish embryo axial mesoderm lineage (3.3h-12h) determined by LTNN(left) and DPT (right).


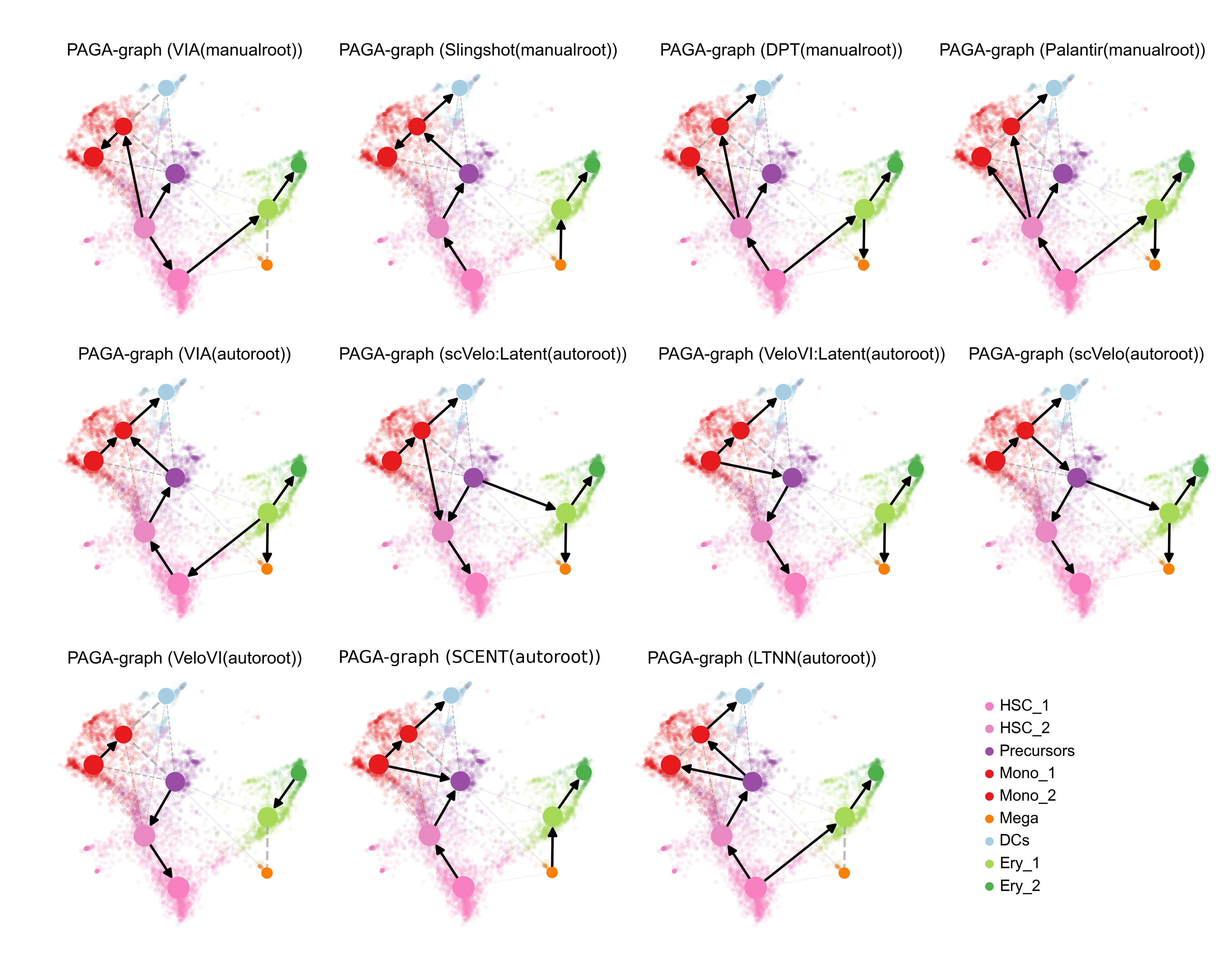


**Figure S4** The direction of cell transition analyzed by different algorithms in human bone marrow cells. The transition direction was predicted by indicated methods was visualized using the PAGA-graph module. Four algorithms - VIA, Slingshot, Palantir, and DPT - with the developmental starting point manually selected, and another five algorithms - VIA, scVelo, VeloVI, SCENT, and LTNN - with the developmental starting point automatically calculated. VeloVI and scVelo computed both velocity-based pseudotime (VeloVI/scVelo) and dynamic model-based pseudotime (VeloVI/scVelo: Latent).


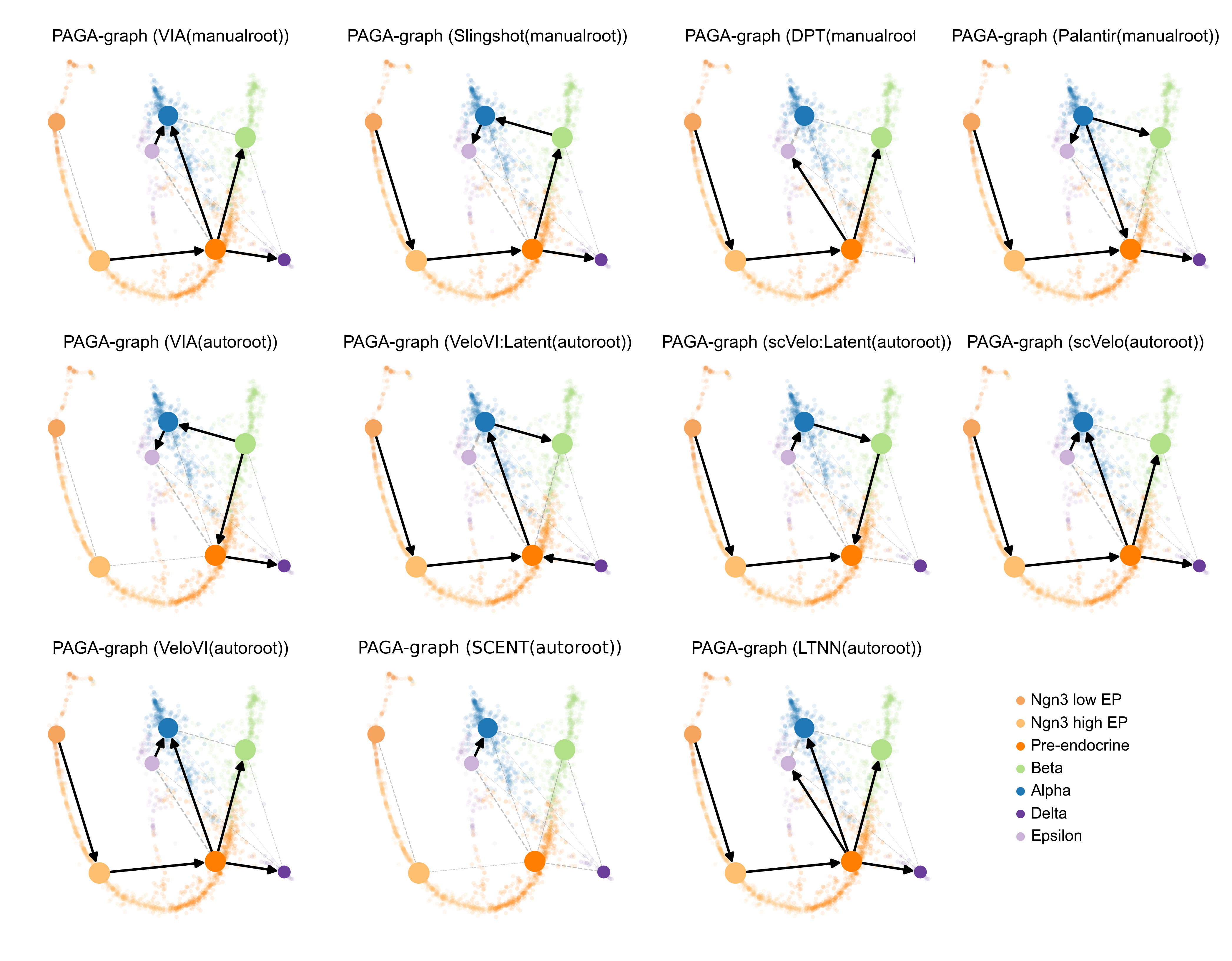


**Figure S5** The direction of cell transition analyzed by different algorithms in human bone marrow cells in mouse pancreatic endocrine lineage. The analysis and data presentation were similar to those described in Figure S4 legend.


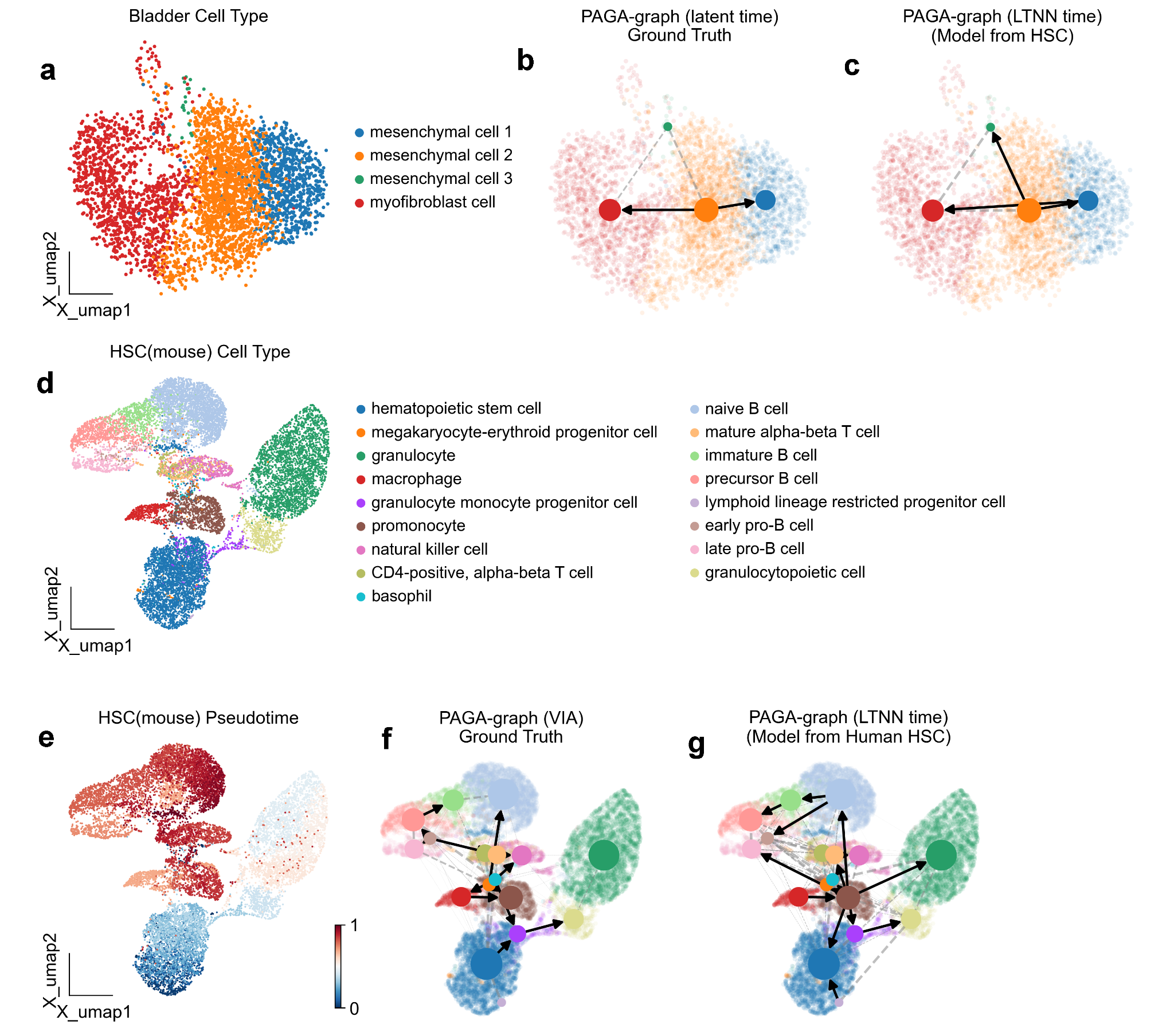


**Figure S6** LTNN's ability to be applied across organs and species. (a) UMAP visualization of human bladder cell types showing distinct clustering of mesenchymal cells and myofibroblasts. (b) PAGA graph illustrating the ground truth latent time trajectory in human bladder cells, highlighting connections between major cell types. (c) PAGA graph depicting the LTNN time of human bladder cells predicted by scLTNN trained with human haematopoietic cell (HSC) data, demonstrating the cross-organ ablitity of scLTNN model. (d) UMAP visualization of mouse bone marrow haematopoietic cell (HSC) cell types, displaying diverse hematopoietic lineages including stem cells, progenitor cells, and differentiated immune cells. (e) Pseudotime analysis of mouse HSCs, indicating the progression of cellular differentiation. (f) PAGA graph showing the ground truth trajectory in mouse HSCs analyzed by VIA, which captured the key lineage bifurcations. (g) PAGA graph showing the mismatched LTNN time using scLTNN model pre-trained with human HSC data, suggesting a limitation of scLTNN model cross-species.
